# Supplementary material for: Effects of Biofilms on Trace Metal Adsorption on Plastics in Freshwater Systems
Source: Int J Environ Res Public Health. 2022 Oct 22;19(21):13752. doi: 10.3390/ijerph192113752 (PMC9658614; doi:10.3390/ijerph192113752)
Supplement: Supplementary file 1 [file ijerph-19-13752-s001.zip › ijerph-1898360-supplementary.pdf]

Supporting information of

Effects of biofilms on trace metal adsorption on plastics in freshwater systems

Zhilin Liu[§1], Tanveer M. Adyel[§2], Jun Wu[1], Zhiyuan Wang*[3], Jianchao Liu[1], Lingzhan Miao*[1], Jun Hou[1]

[1]Key Laboratory of Integrated Regulation and Resources Development on Shallow Lakes, Ministry of Education, College of Environment, Hohai University, Nanjing, People's Republic of China, 210098

[2]Centre for Integrative Ecology, School of Life and Environmental Sciences, Deakin University, Melbourne, VIC 3125, Australia

[3] Nanjing Hydraulic Research Institute, Nanjing, People's Republic of China, 210098

[§] These authors contributed equally to this work.

*Corresponding author:

Professor Lingzhan Miao, Tel.: +86-25-83787930; fax: +86-25-83787930.

Author affiliation: Xikang Road 1st, Nanjing, People's Republic of China, 210098

E-mail: lzmiao@hhu.edu.cn

Text:

Adsorption kinetics

The pseudo-first-order and pseudo-second-order kinetic model, as well as the intraparticle diffusion model were utilized to interpret the kinetics date. Their linear forms as represented by Eqs. (S1)–(S3) are expressed as:

$$\log(Q_e - Q_t) = \log Q_e - \frac{k_1 t}{2.303} \quad (S1)$$

$$\frac{t}{Q_t} = \frac{1}{k_2 Q_e^2} + \frac{t}{Q_e} \quad (S2)$$

$$Q_t = k_t \times t^{\frac{1}{2}} + C \quad (S3)$$

where Q_e (μg/g) and Q_t (μg/g) are the adsorbing contents of heavy metals at equilibrium and at time t (h), respectively; k_1 (h^{-1}), k_2(g/(μg h))and k_t (μg/(g $h^{0.5}$)) represent the rate constants for the pseudo 1st, 2nd order, and for the intraparticle diffusion kinetic models, respectively. Meanwhile, C refers to the constant relationship of the binding layer thickness.

Adsorption isotherm

Langmuir and Freundlich isotherm models were used to study the adsorption behavior of MPs. The Langmuir isotherms model (LM) is often used to describe the homogeneous surface adsorption, while the Freundlich isotherm model (FM) is popularly utilized to describe the enthalpy of heterogeneous surface adsorption. The LM model is represented by Eq. (S4) and the FM model is represented in Eq. (S5):

$$Q_e = \frac{K_L Q_m C_e}{1 + K_L C_e} \quad (S4)$$

$$\log Q_e = \frac{1}{n} \log C_e + \log K_F \quad (S5)$$

where C_e (μg/L) represents the metal ion concentrations and the equilibrium concentration of the heavy metal ions; K_L (L/μg) represents the Langmuir constant. Q_e (μg/g), and Q_m (μg/g) are the adsorption quantity of heavy metals at equilibrium and

the Langmuir monolayer saturation, respectively; K_F (L/μg) is the constant of Freundlich adsorption and 1/n refers to the adsorption intensity.

ICP-MS Analysis Methods:

The samples were introduced into a temperature controlled spray chamber using a MicroMist Nebulizer (Glass Expansion, Pocasset, MA). Helium was used as the collision cell gas to reduce isobaric interferences. Calibration standards of 0.01, 0.05, 0.1, 0.5, 1, 5, 10, 50, 100, 500, and 1000 μg/L were prepared from a CertiPrep ME 2A stock solution (SPEX CertiPrep, Metuchen, NJ) in 3% TraceMetal Grade HNO_3 (Fisher Scientific) in 18.2 MΩ-cm water. The calibration range for Na, Mg, K, Ca, and Fe was extended using instrument standards diluted from a CertiPrep ICS3 Standard to 1000 μg/L with 3% TraceMetal Grade HNO_3 (Fisher Scientific) in 18.2 MΩ-cm water. A stock solution containing Sc, Ge, Y, In, and Bi (Inorganic Ventures, Inc. Christiansburg, VA) was diluted to 15 mg/L in 3% HNO_3 and introduced by peristaltic pump at a 1:30 ratio relative to the sample for use as an internal standard. A NIST Standard Reference Material 1640a (National Institute of Standards and Technology, Gaithersburg, MD) consisting of acidified spring water with mass fractions and concentrations assigned for metal elements was analyzed to assess accuracy. Quality control standards consisting of a ME 2A at a concentration of 50 μg/L ICS3 and 1000 μg/L were analyzed every 10th sample (or more frequently) to check for drift in instrument response.

SEM Analysis Methods:

We followed following steps (1) Fixation: Put 2.5% glutaraldehyde solution into a glass dish and submerge the biofilm sample, store it in a refrigerator at 4°C for 12 hours; (2) Flush: Wash and remove with phosphate buffered saline (PBS) with pH 7.2 Glutaraldehyde, rinse repeatedly three times, each time is 10 minutes; (3) Dehydration: discard the PBS in the container, and then use different concentrations of ethanol solution for gradient dehydration (concentrations of 50, 70, 90, and 100%). Each immersion time is set to 15 min; (4) Drying: put the sample in the freezer of -80℃ refrigerator for 30 min, then put it in a freeze dryer to freeze-dry; (5) sticky sample: apply conductive tape to the sludge sample Stick on the special aluminum sheet for scanning electron microscope; (6) Spray gold: place the glued sample on the ion sputtering coater to coat the surface with a metal film with a thickness of 1000-1500 nm, and place the sample after the gold spray Tested in the sample box at 5.0 kV.

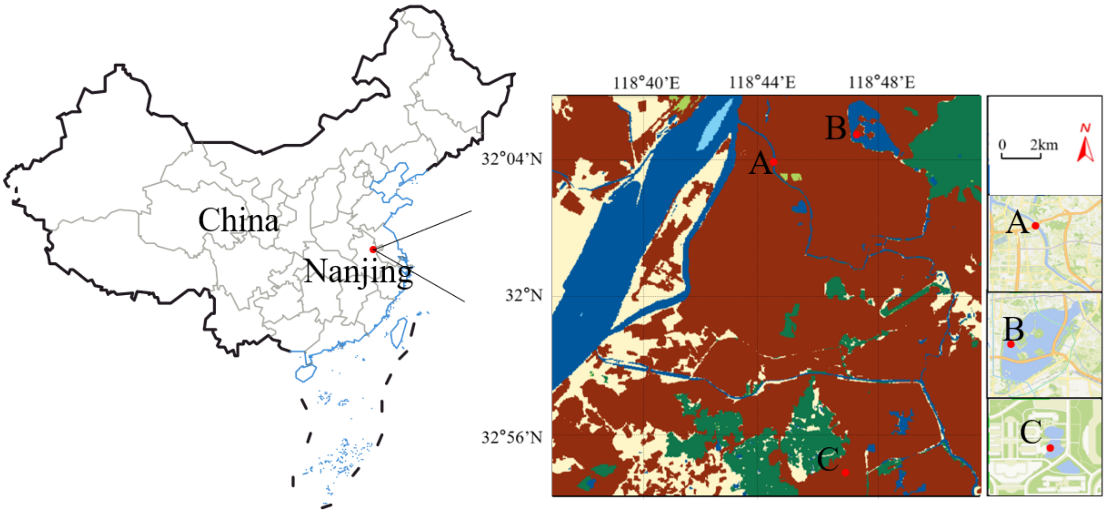

Figure S1. Map of three freshwaters: the Qinhuai River (A), Xuanwu Lake (B) and Donghu Lake (C).

Table S1 Composition of MMS solution*

Component	Concentration (μmol/L)
$CaCl_2·H_2O$	200
$MgSO_4·7H_2O$	140
$(NH_4)_2SO_4$	910
KNO_3	150
$NaHCO_3$	10
KH_2PO_4	5

*: Ionic strength was adjusted to 0.02 mol/L with $NaNO_3$; pH was adjusted to 6.0 with NaOH and HCl.

Table S2 Hydrophobicity of the plastic debris used in the experiments.

Plastic	Shape	Contact angle(θ, °)
PP	sheet	99.54±0.92
PET	sheet	96.87±0.93
PP1	sheet	68.06±9.38
PET1	sheet	42.41±9.52
PP2	sheet	59.26±4.80
PET2	sheet	50.06±2.54
PP3	sheet	74.42±3.98
PET3	sheet	54.72±2.87

The contact angle was measured to characterize the hydrophobicity of substrates. PP and PET: virgin plastic; PP1 and PET1: biofilm-covered plastic in Xuanwu Lake; PP2 and PET2: biofilm-covered plastic in Donghu Lake; PP3 and PET3: biofilm-covered plastic in Qinhuai River.

Table S3. Atomic percentages (%) for the elements on the plastic debris surfaces before metals Adsorption

Plastic	Xuanwu Lake				Donghu Lake				Qinhuai River			
	C 1s	O 1s	Si 2p	O/C	C 1s	O 1s	Si 2p	O/C	C 1s	O 1s	Si 2p	O/C
PP	97.46	1.35	0.12	0.01	97.54	1.35	0.09	0.01	97.78	1.13	0.07	0.01
PET	65.85	32.92	0.13	0.50	63.15	35.56	0.17	0.56	66.84	31.91	0.16	0.48
PP1	76.01	20.45	2.28	0.27	76.45	20.45	1.88	0.27	75.41	21.32	1.76	0.28
PET1	50.72	45.02	2.13	0.89	51.81	42.13	4.53	0.81	53.22	42.77	1.96	0.80
PP2	73.15	24.54	0.96	0.33	72.86	24.16	1.74	0.33	73.76	22.34	1.98	0.30
PET2	51.37	45.48	1.22	0.89	51.21	44.86	2.24	0.88	54.34	41.91	1.86	0.77
PP3	72.18	24.94	1.24	0.35	72.83	23.65	1.46	0.33	71.59	25.44	1.52	0.36
PET3	53.81	40.12	4.32	0.75	52.25	44.24	1.62	0.85	51.21	44.83	1.43	0.88

PP and PET: virgin plastic; PP1 and PET1: biofilm-covered plastic in Xuanwu Lake; PP2 and PET2: biofilm-covered plastic in Donghu Lake; PP3 and PET3: biofilm-covered plastic in Qinhuai River.
